# The Chloroplast Phylogenomics and Systematics of *Zoysia* (Poaceae)

**DOI:** 10.3390/plants10081517

**Published:** 2021-07-24

**Authors:** Se-Hwan Cheon, Min-Ah Woo, Sangjin Jo, Young-Kee Kim, Ki-Joong Kim

**Affiliations:** Division of Life Sciences, Korea University, Seoul 02841, Korea; cheonsh@korea.ac.kr (S.-H.C.); alskdk8445@hanmail.net (M.-A.W.); adidevil@korea.ac.kr (S.J.); gimyoung2@korea.ac.kr (Y.-K.K.)

**Keywords:** plastome evolution, *Zoysia*, small inversion, *rpl3-trnL-UAG* spacer, simple sequence repeats

## Abstract

The genus *Zoysia* Willd. (Chloridoideae) is widely distributed from the temperate regions of Northeast Asia—including China, Japan, and Korea—to the tropical regions of Southeast Asia. Among these, four species—*Zoysia japonica* Steud., *Zoysia sinica* Hance, *Zoysia tenuifolia* Thiele, and *Zoysia macrostachya* Franch. & Sav.—are naturally distributed in the Korean Peninsula. In this study, we report the complete plastome sequences of these Korean *Zoysia* species (NCBI acc. nos. MF953592, MF967579~MF967581). The length of *Zoysia* plastomes ranges from 135,854 to 135,904 bp, and the plastomes have a typical quadripartite structure, which consists of a pair of inverted repeat regions (20,962~20,966 bp) separated by a large (81,348~81,392 bp) and a small (12,582~12,586 bp) single-copy region. In terms of gene order and structure, *Zoysia* plastomes are similar to the typical plastomes of Poaceae. The plastomes encode 110 genes, of which 76 are protein-coding genes, 30 are tRNA genes, and four are rRNA genes. Fourteen genes contain single introns and one gene has two introns. Three evolutionary hotspot spacer regions—*atpB*~*rbcL*, *rps16*~*rps3*, and *rpl32*~*trnL-UAG*—were recognized among six analyzed *Zoysia* species. The high divergences in the *atpB~rbcL* spacer and *rpl16~rpl3* region are primarily due to the differences in base substitutions and indels. In contrast, the high divergence between *rpl32~trnL-UAG* spacers is due to a small inversion with a pair of 22 bp stem and an 11 bp loop. Simple sequence repeats (SSRs) were identified in 59 different locations in *Z. japonica*, 63 in *Z. sinica*, 62 in *Z. macrostachya*, and 63 in *Z. tenuifolia* plastomes. Phylogenetic analysis showed that the *Zoysia* (Zoysiinae) forms a monophyletic group, which is sister to *Sporobolus* (Sporobolinae), with 100% bootstrap support. Within the *Zoysia* clade, the relationship of (*Z. sinica*, *Z japonica*), (*Z. tenuifolia*, *Z. matrella*), (*Z. macrostachya*, *Z. macrantha*) was suggested.

## 1. Introduction

The plant family Poaceae (grasses) consists of 768 genera and 12,074 species [1]. It is a major source of staple foods, making it the most important flowering plant family. Numerous commercial crops are developed from wild species of grasses, such as wheat (*Triticum aestivum*), rice (*Oryza sativa*), maize (*Zea mays*), barley, millet, oats, ryes, etc. In addition to food crops, numerous ornamental and foraging grasses are also cultivated worldwide. Grasses are dominant in agricultural landscapes and in many natural ecosystems around the world [2,3,4,5].

The old classification system of Poaceae recognized two subfamilies: Festucoideae and Panicoideae [6]. However, molecular systematic studies over the last three decades have radically changed its infrafamilial classification system. The current phylogenetic classification system of Poaceae recognizes 12 monophyletic subfamilies and 51 tribes [7,8,9].

The Grass Phylogeny Working Group determined the phylogeny of Poaceae using three chloroplast and three nuclear marker genes. The results revealed that Poaceae is composed of two major clades, one containing the three subfamilies Bambusoideae, Oryzoideae, and Pooideae (BOP clade), and the other containing the six subfamilies Panicoideae, Arundinoideae, Chloridoideae, Micrairoideae, Aristidoideae, and Danthonioideae (PACMAD clade) [2,3,5,10]. The relationships were later clarified to recognize three basal subfamilies—Anomochlooideae, Pharoideae, and Puelioideae [3,9].

The genus *Zoysia* Willd. belongs to the tribe Zoysieae of subfamily Chloridoideae. Subfamilies Chloridoideae and Danthonioideae within the PACMAD clade were found to be a sister group. The subfamily Chloridoideae comprises more than 1,420 species in approximately 140 genera [4,11] and is further classified into five tribes: Centropodieae, Triraphideae, Eragrostideae, Zoysieae, and Cynodonteae [3]. The tribe Zoysieae is sister to tribe Cynodonteae within the subfamily Chloridoideae. The tribe Zoysieae is further divided into two subtribes, Zoysiinae and Sporobolinae [3,12], the former of which is represented by two genera: *Zoysia* and *Urochondra* C.E. Hubb [3].

The genus *Zoysia* consists of 15–18 species, some of which are widely used to create lawns in temperate climates because they can tolerate wide variations in temperature, humidity, salinity, and sunlight [13]. They are also very popular in golf courses, public lawns, burial grounds, and open slopes of mountains owing to their tendency to form dense mats and notably strong tolerances to disease and drought [14]. Several *Zoysia* cultivars have previously been developed through hybridization and selection, and are available commercially as sod or strip. Two popular species—*Z. japonica* and *Z. tenuifolia*—are commonly known as Korean lawn grass, Japanese lawn grass, or oriental lawn grass. Species of *Zoysia* are widely distributed from the temperate regions of Northeast Asia—including Korea, Japan, and China—to the tropical regions of Southeast Asia. Among these, four species—*Z. japonica* Steud., *Z. sinica* Hance, *Z. tenuifolia* Thiele, and *Z. macrostachya* Franch. & Sav.—are naturally distributed in the Korean Peninsula [14].

This study determined the plastome sequences and genome structures of the aforementioned Korean *Zoysia* species, two of which—*Z. japonica* and *Z. tenuifolia*—are used commercially as *Zoysia* grasses, and *Z. sinica* and *Z. macrostachya* are known to be salt-tolerant species [13,14]. Comparative analysis was performed on these four *Zoysia* species and two others, i.e., *Z. matrella* [15] and *Z. macrantha*, for which plastome sequences were previously published. Additionally, phylogenetic analysis using complete gene sequences was performed to establish the relationships between *Zoysia* (Zoysiinae) and other lineages of Poaceae. This study provides genetic information on a useful horticultural plant and will contribute to our understanding of plastome evolution and phylogenetic relationships within the PACMAD clade.

## 2. Results

### 2.1. Chloroplast Genome Structure

NGS sequencing of *Z. tenuifolia* generated 5,385,995 total reads with an average read length of 257.3 bp. A total of 172,448 plastid reads was collected and used for plastid contig assembly. A single plastid contig covering 100% of the plastome was recovered. The complete plastomes of *Zoysia* species were 135,854~135,904 bp long, with an LSC region (81,348~81,392 bp), an SSC region (12,582~12,586 bp), and two IR regions (20,962~20,966 bp). The overall AT base content of the sequences was 61.6% in all four species (Table 1).

**Figure 1:**
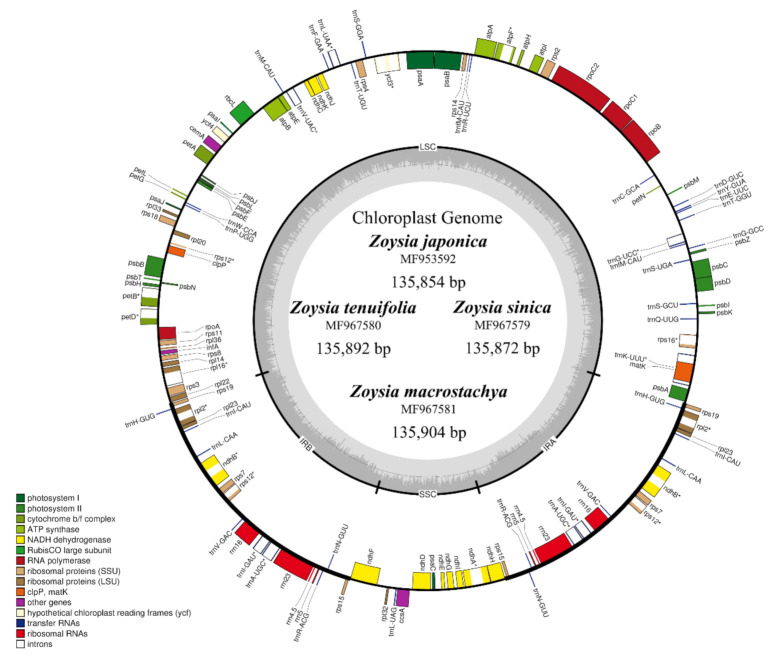
Genome map of the complete chloroplast sequences of *Zoysia japonica* and three other *Zoysia* species sequenced in this study. The gene order and structure among *Zoysia* species are the same. The gray region within the inner circle shows the GC content ratio.

The plastomes encoded 110 unique genes (76 protein-coding genes, 30 tRNA genes, 4 rRNA genes). Fourteen genes contained single introns and one (*ycf3*) had two introns (Figure 1 and Table 2). Unlike in typical angiosperms, the *accD*, *ycf1*, and *ycf2* genes and introns in the *clpP* and *rpoC1* genes were absent.

**Figure 2:**
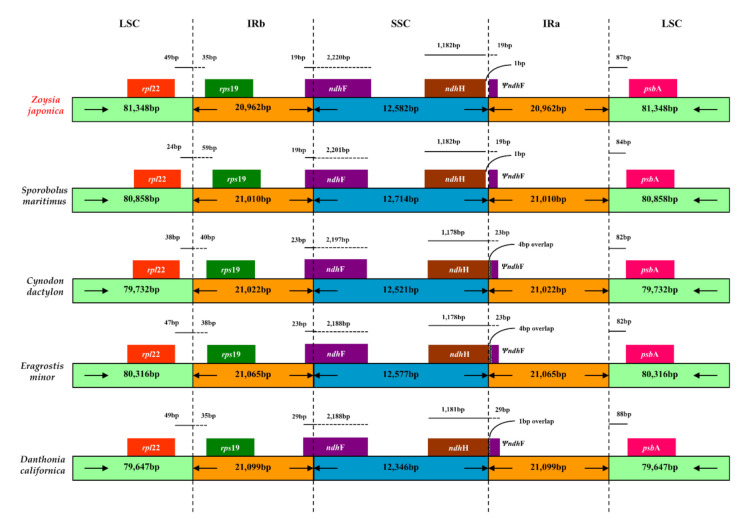
Comparison of the borders of the large single-copy (LSC), small single-copy (SSC), and inverted repeat (IR) regions among the plastomes of five Poaceae species. The IR regions of *Zoysia* species extend into the *ndhF* gene, which is characteristic of the PACMAD clade of Poaceae. Four comparative species were selected from closely related genera to *Zoysia*.

The plastome maps of the four *Zoysia* species are shown in Figure 1. The structural organizations, gene and intron contents, gene order, and AT contents of the plastomes are similar to those of two published *Zoysia* plastomes (*Z. matrella* and *Z. macrantha*) and of typical poaceous plants such as *Sporobolus maritima* [17], *O. sativa* [18], and *Z. mays* [19]. Three plastome sequences from Chlorioideae (*Sporobolus maritima*, *Cynodon dactylon*, and *Eragrostis minor*) and one plastome sequence from Danthonioideae (*Danthonia calyfornica*) were selected, and detailed comparisons of their IR/SC boundaries are presented in Figure 2. Only minor length variations were observed because all species are phylogenetically closely related to each other. The *ndhF* pseudogene at the IR/SSC boundary ranged from 19 to 29 bp long. In addition, the *ndhH* gene and the *ndhF* pseudogene overlapped by 1 bp in the plastomes of *Zoysia* and *Sporobolus*. The IR/LSC boundary is located between *rps19* and *rpl22* in all species. The lengths from the IR/LSC boundary to the *rps19* gene ranged from 35 to 59 bp, depending on the species.

### 2.2. Sequence Divergence in the Zoysia Chloroplast Genomes

The plastomes of five *Zoysia* species (*Z. tenuifolia, Z. sinica*, and *Z. macrostachya* from this study, and *Z. matrella* and *Z. macrantha* from published GenBank sequences) were plotted using the plastome of *Z. japonica* as a reference (Figure S1). The results showed that most of the sequences have high similarity and that the regions of low similarity between *Z. japonica* and the other *Zoysia* plastomes are mainly distributed in the spacer regions of SC regions. Within the LSC region, an intergenic spacer (IGS) between *trnG-UCC* and *trnT-GGU*, the *rpoC2* gene, the IGS from *atpB* to *rbcL*, and the region from the intron of *rpl16* to the *rps3* gene showed notable differences. In the IR and SSC regions, the IGS from *rpl32* to *trnL-UAG* also showed notable differences.

**Figure 3:**
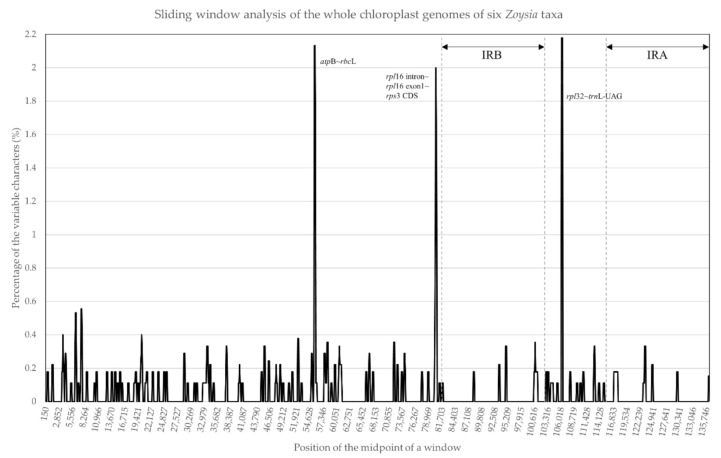
Sliding window analysis of the whole plastomes of six *Zoysia* species. The *x*-axis represents the midpoint position of a window, and the *y*-axis represents the nucleotide diversity of each window. Three noncoding regions (*atpB~rbcL, rpl16~rps3*, and *rpl32~trnL-UAG*) show more than 2% sequence divergences among the six *Zoysia* species.

**Figure 4:**
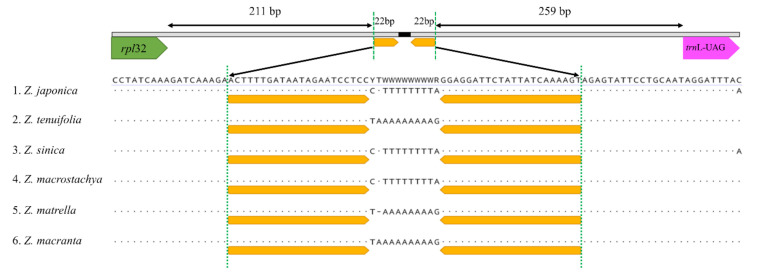
The sequence difference between the *rpl32* and *trnL-UAG* genes among six *Zoysia* species. All six species of *Zoysia* have an 11 bp long loop region, but this region has a different orientation in *Z. japonica*, *Z. sinica*, and *Z. macrostachya* compare to that of *Z. tenuifolia*, *Z. matrella*, and *Z. macrantha*. This difference is due to a small inversion at the 22 bp long stem region (orange color).

Six *Zoysia* plastomes (*Z. japonica, Z. tenuifolia, Z. sinica*, and *Z. macrostachya* from this study, and *Z. matrella* and *Z. macrantha* from published GenBank sequences) were subjected to sliding window analysis (Figure 3). Overall, the percentage of variable characters was observed to be less than 1%, although relatively high values were obtained in three areas. For example, the two parts of the LSC region, *atpB~rbcL* and *rpl16~rpl3* areas, and one part of the SSC region, *rpl32~trnL-UAG* spacer. These three parts showed more than 2% sequence divergences among the six species. The high divergences in the *atpB~rbcL* spacer and *rpl16~rpl3* region are primarily due to the differences in base substitutions and indels. In contrast, the high divergence between *rpl32~trnL-UAG* spacers is due to a small inversion (Figure 4).

### 2.3. Simple Sequence Repeats

Simple sequence repeats (SSRs), also known as microsatellites, which are made up of the same nucleotide sequence or sequence units repeating over a region greater than 10 bp, were analyzed. SSRs were identified in 59 different locations in *Z. japonica*, 63 in *Z. sinica*, 62 in *Z. macrostachya*, and 63 in *Z. tenuifolia* plastomes (Table 3). The major SSR types were homopolymers (*Z. japonica* (50/59), *Z. sinica* (54/63), *Z. macrostachya* (53/62), and *Z. tenuifolia* (54/63)), whereas there were eight dipolymers and one tripolymer in the four *Zoysia* species. Among the homopolymers, two were composed of G or C bases and the rest comprised multiple A or T bases. Dipolymers were composed of AT, GA, TA, or TC units, whereas the single tripolymer was AAT.

### 2.4. Phylogenetic Analysis

**Figure 5:**
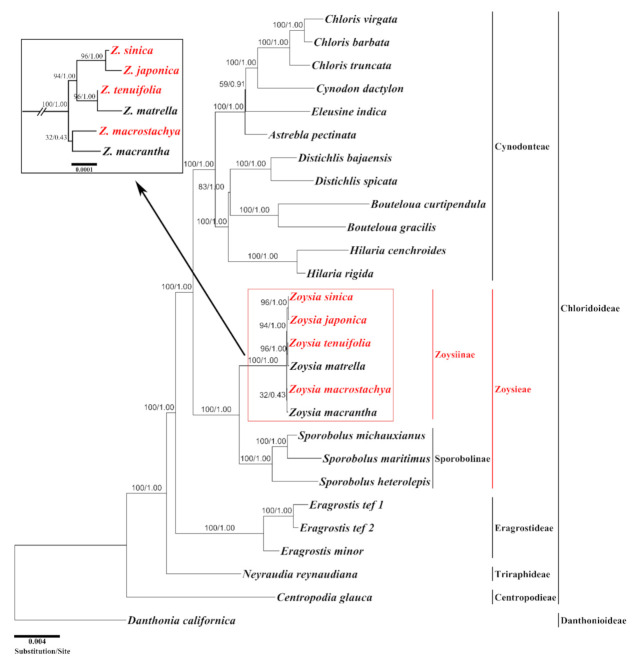
A maximum likelihood (ML) tree constructed from the plastomes of 26 species within the subfamily Chloridoideae (Poaceae) with *Danthonia californica* as an outgroup. The numbers at each node indicate the ML bootstrap values/Bayesian posterior probability. The ML tree was constructed from 76 protein-coding and 4 rRNA genes by RaxML. The newly sequenced species in this study are indicated in red. The short internal nodes among *Zoysia* species are expanded in the box at the upper left corner.

To estimate the phylogenetic relationships in *Zoysia*, an ML tree was constructed from 26 complete plastomes of the subfamily Chloridoideae and one outgroup. *Danthonia californica* of the subfamily Danthonioideae was selected as an outgroup because the two subfamilies are sister groups [20]. The aligned length of 76 protein-coding genes and 4 rRNA genes was 61,097 bp. The ML tree was constructed with an ML optimization likelihood value of −ln = −142,175.088555. The bootstrap support percentages and Bayesian posterior probability values were also given at all internal nodes (Figure 5). The tree recognized five tribes within the subfamily Chloridoideae: Centropodieae, Triraphideae, Eragrostideae, Zoysieae, and Cynodonteae. Two monophyletic subtribes—Zoysiinae and Sporobolinae—were identified in the tribe Zoysieae and formed a sister group. The monophyly of five tribes within the subfamily Chloridoideae, and two subtribes within the tribe Zoysieae were supported by 100 bootstrap percentages and 1.0 Bayesian posterior probability values. All six species of *Zoysia* formed an independent monophyletic group, as did all three species of *Sporobolus*. Within the genus *Zoysia*, three sister species pairs—*Z. sinica—Z. japonica*, *Z. tenuifolia—Z. matrella*, and *Z. macrostachya—Z. macrantha*—were supported by 96%, 96%, and 32% bootstrap support, respectively. The first two species pairs were monophyletic, with 94% bootstrap support. Therefore, the relationships were as follows: (*Z. sinica*, *Z. japonica*), (*Z. tenuifolia*, *Z. matrella*), (*Z. macrostachya*, *Z. macrantha*).

## 3. Discussion

The longest plastome in *Zoysia*—belonging to *Z. macrostachya*—is only 82 bp longer than the shortest, *Z. matrella* (Table 1). This small range of difference is due to sporadic indels at some IGS regions (Figure S1). With the exception of small size differences, there are no noticeable differences among the plastomes of *Zoysia* (Figure 1).

Unlike the genomes of typical angiosperms [21,22,23,24], those of *Zoysia* lack the genes *accD*, *ycf1*, and *ycf2* and introns in the *clpP* and *rpoC1* genes, which is a pattern observed in most grasses [25,26,27]. The loss of two introns in the *clpP* gene has been reported independently in different angiosperm families, including Poaceae [28,29,30], the IR-lacking clade of Leguminosae [31], Oleaceae (opposite-leaved *Jasminum* and *Menodora*) [32], and Onagraceae (*Oenothera*) [33].

Similar to the chloroplast genomes of other grasses, the IR regions in *Zoysia* species extend to the *rps19* gene region (Figure 2). The IR regions of the *Zoysia* plastomes are also extended to duplicate a part of the 5′ end of the *ndhF* gene, a feature that is similar to other poaceous plastomes [19,34,35,36]. Our results indicate that this extension of the IR into the *ndhF* gene is characteristic of the PACMAD clade but not the BOP clade. In BOP clade species, such as those in the genera *Oryza*, *Agrostis*, *Hordeum*, and *Bambusa*, the IR is extended into a part of the *ndhH* gene, rather than the *ndhF* gene; this is a specific feature of BOP clade members [36,37,38]. Our results also show that *Zoysia* has all the same types of missense mutations and insertions in *rpl16*, *rpl32*, *rpoC1* as *Neyraudia* [39].

It is well known that noncoding regions exhibit a higher level of sequence divergence than do coding regions, and in the present study, we detected relatively high sequence divergences within the noncoding regions of *Zoysia* plastomes (Figure 3). Three noncoding regions—the *atpB*~*rbcL* spacer, *rps16* intron, and *rpl21*~*trnL-UAG* spacer—show more than 2% sequence differences, making them good candidates for plastome markers to differentiate *Zoysia* species. As described above, the *atpB*~*rbcL* spacer and *rps16* intron/spacer regions have high divergences due to the indels and base substitutions, while the high divergence of the *rpl21*~*trnL-UAG* spacer region is primarily due to a small inversion (Figure 4). A small inversion between the 22 bp stem region generated 11 bp differences in the loop region. The orientation of this loop region in three species of *Zoysia* (*Z. japonica*, *Z. sinica*, and *Z. macrostachya*) is different from that of three other species (*Z. tenuifolia*, *Z. matrella*, and *Z. macrantha*). This kind of small inversion is also reported in Poaceae and other flowering plant families [24,35,40].

In addition to the genome research described earlier [15,39], there have been some systematic studies of *Zoysia* using molecular-based markers, such as restriction fragment length polymorphisms (RFLP) [41] and simple sequence repeats (SSR) [42,43]. In the present study, we conducted maximum likelihood analysis based on 76 protein-coding genes and 4 rRNAs using the complete chloroplast genomes and revealed that five tribes—Centropodieae, Triraphideae, Eragrostideae, Zoysieae, and Cynodonteae—are monophyletic groups in the subfamily Chloridoideae. In addition, the tribes Cynodonteae and Zoysieae are sister groups. These relationships are consistent with those established in previous studies [3,11,12,44,45]. Our tree indicates that the subtribe Sporobolinae is sister to the subtribe Zoysiinae (Figure 5). This relationship is also supported by other studies, even though the previous studies only used one representative species of *Zoysia* [44]. We used the six *Zoysia* species to construct the tree, but the same tree topology was generated regarding the sister group relationship of two subtribes. The subtribe Zoysiinae consists of two genera, *Zoysia* and *Urochondria* [3], but plastome sequences are not available from *Urochondria*. The monophyly of genus *Zoysia* was supported by 100% bootstrap support and 1.00 Bayesian posterior probability (BP). The three species pairs—*Z. macrostachya–Z. macrantha*, *Z. tenuifolia–Z. matrella*, and *Z. japonica–Z. sinica*—are all sister in our ML tree, with 32% (0.43 BP), 96% (1.00 BP), and 96% (1.00 BP) bootstrap support, respectively. In addition, the monophyly of the *Z. tenuifolia–Z. matrella* and *Z. japonica–Z. sinica* pairs is also supported by 94% (1.00 BP) bootstrap support. These results are different from those of a previous systematic study [43], in which *Z. matrella* and *Z. japonica* form a monophyly with 68.2% support, and *Z. tenuifolia* is sister to this clade with 100% support. Accordingly, we will need to analyze more complete plastomes to resolve these differences. Whole-genome sequences will provide more convincing evidence for the relationships among the species of *Zoysia* that show low levels of plastome sequence divergence.

## 4. Materials and Methods

### 4.1. Plant Materials, DNA Extraction, and Sequencing

The leaves of *Zoysia tenuifolia* were collected from a greenhouse at the Korea University, where the authors grow plants originally collected from natural habitats in Korea. The leaves of *Z. japonica*, *Z. sinica*, and *Z. macrostachya* were collected directly from plants growing in natural habitats in Korea. Voucher specimens of each species were deposited in the Korea University Herbarium (*Z. tenuifolia*: KUS2017-0002, *Z. japonica*: KUS2013-0161, *Z. sinica*: KUS2010-1956, and *Z. macrostachya*: KUS2013-0307). Fresh leaves were ground into powder in liquid nitrogen, and total genomic DNAs were extracted using the CTAB method [46]. The DNA was purified using CsCl/EtBr gradient ultra-centrifugation [47], then further purified by dialysis [48]. The genomic and chloroplast DNAs were deposited into the Plant DNA Bank in Korea (PDBK) under the accession numbers PDBK2017-0002, PDBK2013-0161, PDBK2010-1956, and PDBK2013-0307.

Approximately 100 ng of *Z. tenuifolia* DNA was used for NGS sequencing using an Ion Torrent PGM™ sequencer (Thermo Fisher Scientific, Waltham, MA, USA). The library was fabricated as pair ends through an Ion Plus fragment library kit (Thermo Fisher Scientific, Waltham, MA, USA). The target library size was 300 base reads, and fragmentation was performed using a Bioruptor^®^ UCD-600 NGS Sonication system (Diagenode, Denville, NJ, USA). The library size selection was performed using E-gel^®^ size select™ 2% Agarose gel (Thermo Fisher), and Bioanalyzer^®^ instrument analysis; 2100 Bioanalyzer (Agilent Technologies Inc., Santa Clara, CA, USA) was used for library normalization/quantification. Ion PGM™ Sequencing 400 Kit and Ion 318™ Chip v2 (Thermo Fisher Scientific) were used for sequencing. The raw sequenced NGS data were uploaded to the National Center for Biotechnology Information (NCBI) Sequence Read Archive (SRA; acc. No. PRJNA548200).

NGS sequencing was performed on *Z. tenuifolia* and PCR sequencing was performed on the other three—*Z. japonica*, *Z. sinica*, and *Z. macrostachya*. Purified total DNAs of *Z. japonica*, *Z. sinica*, and *Z. macrostachya* were amplified using a series of primer sets developed from *the Z. tenuifolia*, *Z. matrella*, and *Panicum virgatum* [49] plastomes. The primers used to sequence the three *Zoysia* species are listed in Tables S2–S4. Continuous sequencing was performed on PCR products using primer walking for whole-plastome sequencing [50]. The PCR products were purified using a MEGAquick-spin Total Fragment DNA purification Kit (iNtRON Biotechnology, Seongnam, Korea), and the cleaned products were sequenced in both directions using an ABI 3730xl DNA analyzer (Thermo Fisher Scientific, Waltham, MA, USA).

### 4.2. Chloroplast Genome Assembly and Annotation

All of the generated raw reads were trimmed using a basic application in Geneious version 8.1.9 [51] with an error probability limit = 0.05. We assembled the trimmed raw reads with *Z. matrella* as the reference. After mapping the reads to the plastome of *Z. matrella*, low-coverage regions were removed. Trimmed reads were then mapped again to the obtained contigs to form longer contigs. After removing the low-coverage regions again, the contigs with overlapping regions were merged to create larger contigs. This process was repeated for the entire plastome sequence.

Initial annotations of the four chloroplast genomes were generated by DOGMA [52]. To complement the out-of-date DOGMA database, these annotations were manually edited by comparing them to published Poaceae chloroplast genomes (Table S1) using BLAST. When a discrepancy was found, the annotation was confirmed using the ORF finder program from the National Center for Biotechnology Information (NCBI) and tRNAscan-SE 2.0 [53] for the corresponding parts. Circular chloroplast genome maps were generated using OrganellarGenomeDraw [54].

### 4.3. Sequence Analysis

Two previously published *Zoysia* plastome sequences—*Z. matrella* (GenBank Acc. No.: AP014937) and *Z. macrantha* (GenBank acc. No. KT168390)—were included in our sequence comparison analysis. To plot sequence divergence among the *Zoysia* chloroplast genomes, we used mVISTA [55] with default parameter settings. The plastome sequences were aligned using MAFFT [56] and sliding window analysis was performed using DnaSP v6 [57] on the aligned sequence. The window length was set to 300 bp, with a 100 bp step size. Simple sequence repeats (SSRs) were analyzed using the Phobos tandem repeat tool [58] in Geneious v8.1.9 [51] with default parameters.

### 4.4. Phylogenetic Analysis

We constructed the phylogenetic tree using whole plastid sequences from all available plastome sequences of subfamily Chloridoideae and a member of subfamily Danthenoideae, *Danthonia californica*. A total of 27 complete chloroplast genomes were used for phylogeny construction (Table S1). The 76 protein-coding gene and four rRNA gene sequences were extracted from each plastome and then aligned using MUSCLE v.3.8.425 [59] with default parameters. We selected the GTR base substitution model based on the Akaike Information Criterion (AIC) through the PAUP modeltest [60]. Maximum likelihood (ML) analysis was conducted using RAxML-HPC BlackBox v.8.2.10 [61] in CIPRES Science Gateway v3.3.3 [62]. Default parameters were used, and the ML bootstrap support values of each internal node were evaluated using 1000 replications. Bayesian posterior probability was conducted using MrBayes v3.2.6 [63] plugin tool in Geneious v8.1.9 [51]. The substitution model was GTR and the rate variation was set to equal. The MCMC chain length was 1,000,000 and the subsampling frequency was 100. The heated chain was set to 4, burn-in length to 2500, and heated chain temperature to 0.2.

## 5. Conclusions

In this study, we identified four new plastome sequences from species of *Zoysia* and described the evolutionary features of these genomes. There was no significant difference among these plastomes. In addition, we examined phylogenetic relationships using all the chloroplast genes of six *Zoysia* species. The genus *Zoysia* formed a monophyletic group and the tribe Zoysieae consisted of the subtribes Sporobolinae and Zoysiinae. However, analysis of a larger number of plastome sequences will be needed to more accurately determine the phylogenetic relationships among *Zoysia* species.

## Figures and Tables

**Table 1:** The comparative feature of six *Zoysia* plastomes.

Species	*Zoysia japonica*	*Zoysia tenuifolia*	*Zoysia sinica*	*Zoysia macrostachya*	*Zoysia matrella* *	*Zoysia macrantha* *
Accession number	MF953592	MF967580	MF967579	MF967581	AP014937	KT168390
Genome length (bp)	135,854	135,892	135,872	135,904	135,810	135,845
LSC length (bp)	81,348	81,376	81,366	81,392	81,308	81,352
SSC length (bp)	12,582	12,584	12,582	12,586	12,583	12,576
IR length (bp)	20,962	20,966	20,962	20,963	20,960	20,959
AT content	61.6%	61.6%	61.6%	61.6%	61.6%	61.6%
Total gene contents	110	110	110	110	110	110

* Indicates previously published sequences [15,16] available from the NCBI database.

**Table 2:** Genes in the *Zoysia* plastomes. Each species has 110 unique genes.

Genes Category	Group of Genes	Name of Genes
Self-replication	rRNA genes	*rrn16* (x2), *rrn23* (x2), *rrn4*.5 (x2), *rrn5* (x2)
	tRNA genes	30 *trn* genes (8 are in IR regions)
	Small subunit of ribosome	*rps2*, *rps3*, *rps4*, *rps7* (x2), *rps8*, *rps11*, *rps12* * (x2), *rps14*, *rps15* (x2), *rps16* *, *rps18*, *rps19* (x2)
	Large subunit of ribosome	*rpl2* * (x2), *rpl14*, *rpl16* *, *rpl20*, *rpl22*, *rpl23* (x2), *rpl32*, *rpl33*, *rpl36*
	DNA dependent RNA polymerase	*rpoA*, *rpoB*, *rpoC1*, *rpoC2*
Genes for photosynthesis	Subunits of NADH-dehydrogenase	*ndhA* *, *ndhB* * (x2), *ndhC*, *ndhD*, *ndhE*, *ndhF*, *ndhG*, *ndhH*, *ndhI*, *ndhJ*, *ndhK*
	Subunits of photosystem 1	*psaA*, *psaB*, *psaC*, *psaI*, *psaJ*
	Subunits of photosystem 2	*psbA*, *psbB*, *psbC*, *psbD*, *psbE*, *psbF*, *psbH*, *psbI*, *psbJ*, *psbK*, *psbL*, *psbM*, *psbN*, *psbT*, *psbZ*
	Subunits of cytochrome b/f complex	*petA*, *petB* *, *petD* *, *petG*, *petL*, *petN*
	Subunits of ATP synthase	*atpA*, *atpB*, *atpE*, *atpF* *, *atpH*, *atpI*
	Large subunit of rubisco	*rbcL*
Other genes	Maturase	*matK*
	Protease	*clpP*
	Envelope membrane protein	*cemA*
	c-type cytochrome synthesis gene	*ccsA*
	Translational initiation factor	*infA*
Genes of unknown functions Open Reading Frames (ORF)		*ycf3* **, *ycf4*

* One or ** two asterisk(s) beside the gene name indicate(s) that the gene contain(s) one or two intron(s), respectively. “(x2)” indicates that the gene is located on the IR regions.

**Table 3:** Distribution of simple sequence repeats (SSRs) among the plastomes of *Zoysia japonica*, *Z. sinica, Z. macrostachya*, and *Z. tenuifolia*.

Unit	Length (bp)	*Zoysia japonica*	*Zoysia sinica*	*Zoysia macrostachya*	*Zoysia tenuifolia*
		No. of SSRs	No. of SSRs	No. of SSRs	No. of SSRs
A	17	0	1	0	0
	16	2	1	1	3
	15	1	0	0	1
	14	2	3	0	1
	13	2	2	3	3
	12	3	3	6	3
	11	4	4	6	6
	10	10	9	7	9
C	11	1	1	0	0
	10	0	0	0	1
G	11	0	0	0	1
	10	1	1	2	0
T	22	1	0	0	1
	17	0	4	0	0
	16	0	0	1	0
	14	0	0	2	1
	13	3	4	4	4
	12	7	7	5	7
	11	5	6	4	7
	10	8	8	12	6
AT	12	1	1	1	1
	10	3	3	3	3
GA	10	1	1	1	1
TA	14	1	1	1	1
	10	1	1	1	1
TC	10	1	1	1	1
AAT	12	1	1	1	1
Total		59	63	62	63

## Data Availability

The chloroplast sequence data presented in this study are available in GenBank (NCBI acc. nos. MF953592, MF967579~MF967581). The raw sequenced NGS data were uploaded to the National Center for Biotechnology Information (NCBI) Sequence Read Archive (SRA; acc. No. PRJNA548200).

## Supplementary Materials

Supplementary Materials can be found at https://www.mdpi.com/article/10.3390/plants10081517/s1. Figure S1: mVISTA identity plot comparing *Zoysia* plastomes. The *Z. japonica* plastome was used as a reference. The vertical scale indicates the percentage identity, ranging from 50% to 100%. White, blue, and pink regions indicate unmatched, coding, and noncoding regions, respectively, Table S1: The GenBank accession number used in this study, Table S2: The Primer list of sequencing of *Zoysia japonica*, Table S3: The Primer list of sequencing of *Zoysia macrostachya*, Table S4: The Primer list of sequencing of *Zoysia sinica*.

## Abbreviations

LSC	Large Single Copy
SSC	Small Single Copy
IR	Inverted Repeat
SSR	Simple Sequence Repeat
ML	Maximum Likelihood
