# Supplementary material for: The Chloroplast Phylogenomics and Systematics of Zoysia (Poaceae)
Source: Plants (Basel). 2021 Jul 24;10(8):1517. doi: 10.3390/plants10081517 (PMC8400354; doi:10.3390/plants10081517)
Supplement: Supplementary file 1 [file plants-10-01517-s001.zip › plants-1272299-supplementary/Table S1. The GenBank accession number used in this study.pdf]

Table S1. The GenBank accession number used in this study

GenBank accession Number	Species
KT168391	*Astrebla pectinata*
KT168386	*Bouteloua curtipendula*
KT168392	*Bouteloua gracilis*
KT168383	*Centropodia glauca*
KT168393	*Chloris barbata*
KX765278	*Chloris truncata*
KX765279	*Chloris virgata*
KY024482	*Cynodon dactylon*
KJ920229	*Danthonia californica*
KT168394	*Distichlis bajaensis*
KT168395	*Distichlis spicata*
KU833246	*Eleusine indica*
KT168384	*Eragrostis minor*
KT168385	*Eragrostis tef 1*
MF035981	*Eragrostis tef 2*
KT168387	*Hilaria cenchroides*
KT168396	*Hilaria rigida*
KF356392	*Neyraudia reynaudiana*
KT168389	*Sporobolus heterolepis*
KP176438	*Sporobolus maritimus*
KT168388	*Sporobolus michauxianus*
KT168390	*Zoysia macrantha*
AP014937	*Zoysia matrella*
MF953592	*Zoysia japonica*
MF967579	*Zoysia sinica*
MF967580	*Zoysia tenuifolia*
MF967581	*Zoysia macrostachya*
