# Supplementary material for: The Chloroplast Phylogenomics and Systematics of Zoysia (Poaceae)
Source: Plants (Basel). 2021 Jul 24;10(8):1517. doi: 10.3390/plants10081517 (PMC8400354; doi:10.3390/plants10081517)
Supplement: Supplementary file 1 [file plants-10-01517-s001.zip › plants-1272299-supplementary/Table S2. The Primer list of sequencing of Zoysia japonica.pdf]

Table S2. The Primer list of sequencing of *Zoysia japonica*

Primer ID	Sequence(5'-3')	Strand*	Position
**320**	TAGCCTTTGTTTGGCAAGCT	-	7389
**395**	CTTCGGGATCGAACATCAAT	-	4643
**petB**	CGTCTTGAGATTCAGGCAATTGCG	-	72915
**385**	ACCTCCGGAGCTAATGAAAC	+	78020
**381**	TATTCAGGGGATCCTTTCCC	-	78630
**rpoC1_1**	GTGGATACACTTCTTGATAATGG	+	24530
**rpoC1_4**	CCAACTCAAGATATGCTTATGG	-	25127
**rps16**	GTTCTTGTAGGTTGAGCGCCTTT	-	4529
**trnK5**	TACTCTACCRTTGAGTTAGCAAC	+	3902
**22**	GTTCCTGTAGGTTGAGCCCCTTTTTCAAGG	-	4529
**23**	TTAAAAGCCGAGTACTCTACCGTTGAGTTA	+	3890
**301**	ATCCGTTACGGTCGTCATTC	+	44167
**w54225f**	TCCTGAAGTTCTTTGTAACGTTGT	+	53720
**w55203**	ACAGCTCTAACTCGATTATTTCCT	+	54698
**w56135**	AGACCCTGTCGTTGTGAGAA	+	55686
**ccsAf**	TTAGAACATATACTAACTCA	+	106187
**ccsAr**	TAATTTGGATCTGCTATTTT	-	107058
**284**	AAAAGAACCGGCACAATACG	-	129223
		+	87960
**259**	AAAAACCAAACTCTGCCTTACG	+	126586
		-	90596
**40**	CCTTGGCTAAACCTTGGAAATCGTAATGC	-	40118
**370**	GCAAGGCCTAATTGAAAAATGA	+	39140
**247**	TCTCCGTTCCAGAACCGTAC	-	44199
**221**	CAACCAATTATGCGCTTCAATATAATTACCAGGA	+	43084
**99**	GTAGATGCTACATAGTTGGTTCTCAT	+	129267
		-	87911
**106**	TTGGGTTCATTGATATTCCTGGTATAG	+	86789
		-	130388
**182**	GAAGGTCACGGCGAGACGAG	+	99278
		-	117906
**9**	GCCGACCGCTCTACCACTGAGCTACTGAGG	+	116828
		-	100346
**339**	AACTGCAAAATCCGATCCAA	+	127461
		-	89723
**14**	TAGCCCCAGAATGTTTCAGTTTATGCTCCT	-	128662
		+	88512
**165**	CATTACCAAGATTAGCACGG	+	165
**168**	TGCGTTTGTGGGTGGAATCCCCCGAG	-	39354
**18**	GTCTGTAAATACTGCATATTTGATTCCATC	+	83668
		-	133506
**340**	TTGGAACGAGCATGAAGAAA	-	84924
		+	132260
**71**	GTTGCTTTCTACCACATCGTTTCAAACGAA	+	5525

**72**	CCCGTTGCCTTACCACTTGGCCACGCCCCA	-	6862
**27239F**	CACTCATGGGGATCTGGT	+	26730
**29456R**	CAATACCAATTCCTTTTATTCCA	-	28968
**29252F**	TCAATTCGTTCGAACTTGCTT	+	28764
**31653R**	TATGACGAGGTTGCCTGACA	-	31180
**13**	GTAACGCGTAAGAACCTGCCCTTGGGAGGGG	-	92722
		+	124451
**393**	TCCGAATCCGCTTTGTCTAC	+	92569
		-	124615
**43**	GCCC-ACTATTGGCCAAACCACCTGGGCACTAG	+	42067
**90**	TCCAGGCTTATTTGTCAAAAAAAAATACACT	+	102112
		-	115061
**8**	GAACCACGGTGTCGGGGGTTCGAATCCCTC	+	100014
		-	117160
**303**	TAGGTTCGAATCCTACTTGGG	-	100308
		+	116875
**39**	ACCTTATATTCCCTGAGGAGGTTCTACCACGT	+	10042
**38**	TTGAAGAATCTTTTCCCTTCTTTGGTTATGTC	-	10454
**25**	CTGGGACGGAAGGATTCGAACCTCCGAATAGC	+	6820
**37**	ACTGTAAATTCGTTGGCAATATGTCTACGC	-	15660
**161**	GCCCTTTTAACTCAGTGGTAGAC	+	14882
**148**	GGCTTATATAGGTGAATCCTAGGA	+	32378
**145**	GATTGGCTGTAGGGCTTGCTTCT	-	33267
**321**	TGCTTAGTCTAGCTTTTATGGAAGC	-	33387
**384**	GAGGGGAAGGATGGATTGTT	+	10691
**164**	CTTAAAACTATACCGGTTTTCAAGA	-	11646
**188**	TCAACCACTCAGCCATCTCTCCG	-	11680
**246**	CCTTCCCTTAGAACCGTACTTG	-	43229
**218**	TTCCTTCCCTTAGAACCGTACTTGAGAG	-	43227
**73**	CGGAAAGAGAGGGATTCGAACCCTCGGTACG	+	8059
**368**	GTTGGTGTCAATTAGGCGGT	-	9337
**76**	AATCCACTGATTTCTGCCGCTTCCGTTATTGC	+	33230
**356**	TGCTGAATCGACGACCTATG	-	34490
**24**	CGCCGCTTTAGTCCACTCAGCCATCTCTCC	-	8117
**15**	GCTGTTAATACGAATAGAGAAACTCTGT	-	88595
		+	128580
**19**	GTTGGGCGCTTTAACCATTCAGCCATGGATGC	-	83917
		+	133255
**H415**	TATGGGGATGGTGAGAAGAG	-	134082
		+	83102
**H428**	AAGGGGCATGATGACTTGAC	+	123423
		-	93761
**H427**	ACCTTGACGTGGTGGAAGTC	+	92347
		-	124837
**H429**	CGCATCTTCACAGACCAAGA	+	94819
		-	122365

**97**	ATATAGCTCAGTTGGTAGAGCTCCG	-	95268
		+	121911
**102**	CGCTCTACCACTGAGCTAATAGCCC	-	122758
		+	94421
**H426**	CCCTATTGTTCCGATGGAGA	+	128422
		-	88762
**H424**	TTTCGACCTTGGTTCCGTAG	+	87764
		-	129420
**H422**	CTAAAGAGCGTGGAGGTTCG	-	131057
		+	86127
**11**	CATTTCGCTCGCCGCTACTACGGGAATCGC	+	120664
		-	96510
**21**	AACAGCAATCCAAGGTCGCATACCCAGACGGA	+	715
**W4052**	CAAAATAAACAGATCGGT	+	4059
**48**	CCCCAGAACCAGAAGTAGTAGGATTGATTCT	-	54894
**W-2**	TTCTCGCTATTTGGAATGGA	-	84119
		+	133065
**W90942**	AATAAGGTTTGATCCTATTCATGG	+	128104
		-	89076
**256**	CTGGGGAGGTCCGTTTGATA	+	134992
		-	82192
**W-3**	AATCCACTGCCTTGATCCAC	-	135373
		+	81811
**W3004**	TGCTGATACATTAGAAAAATTAAA	+	3015
**W7487**	TGGATCTGTGCGGTAAGGAAAA	+	7762
**W27367**	CCGACCTTGTACGATCCC	+	26858
**W108608**	AAGCACAATAACTACGCCAAG	+	106485
**W110557**	TAATTCTAACTCCCACATGATAAAA	-	108402
**W112176**	CGATTCATTTCAATCTGAGGA	+	110020
**W114051**	TTCGGTTTGATAACCTGCTA	-	111953
**W-1**	CGAGATCCACCCTACAATA	+	127330
		-	89855
**W6509**	TTTTGTGTCGTGCAGAGC	+	6770
**H465**	AGTGGTTCAAGGCGTAGCAT	-	45638
**210**	GTGTGTATTTTTCGTTGGGGATTT	-	58812
**288**	ATCAATATATCTGTTATGTAGCTTACCCCTTAGAC	+	56020
**129**	GCTCTGTAGGATTTGAACCTACGACATC	+	64771
**237**	AAGTATTTTACGATTAAGAAG	-	67258
**264**	CCTTTTTCATTGCTTTTCGA	-	80331
**W113787**	TTGATCCATTCTCATCCTGG	+	111689
**W115436**	CAAAACGATATCCCAAAGGG	-	113345
**212**	CAGGAGCAGGGTCGGTCAAAT	-	53910
**C011**	GGGCTATACGGACTCGAACC	+	51987
**374**	CATCTCGGAAATATTCAGCCA	-	54141
**82**	CTATGGAGAAATTGGTAGACACGCTGCTCT	-	106020
**C002**	GACCCCATACCTAGAGCTAACA	+	103499

**H409**	GGTGAAATTGGTAGACACGC	-	106024
**W108711**	TCTCAGTGGTTAATGATGCATG	-	106568
**W947**	GAAACAGGCTCACGAATACCA	+	947
**W2279**	GCGAGAAATTGACAAGGTAAGA	-	2290
**W4463**	TCAAAGTTCTTTCTTTATCTTTAAA	+	4473
**W5430**	CTATGTCGAGCCAAGAGCATT	-	5441
**H481**	AATGATGTCGAGCCAAGAGC	+	5439
**W7136**	GCGTTTGTTTGGCAAGC	+	7391
**163**	AGGAGAGAGAGGGATTCGAACCCT	+	11614
**W13690**	AGAACGAATCACACTTTTACCAC	-	13839
**162**	CATCGGTTCAAATCCGATAAGGGGCT	-	14928
**W13308**	CGGTTCTAGTAAACCAAAACCA	+	13458
**W25288**	GCTTTTTCAGCTATTTGTAATTCG	+	24748
**W27428**	CACACGTACCCGTCATGG	-	26919
**W29987**	TCAAATTTTCATTGTTAATATGGAC	+	29505
**W31365**	TTTTGATGCAGCAAGTCAGG	+	30892
**146**	GCAGCATGGTACTCTTTCTACCACTA	+	34319
**W37015**	AAAGCCATGAAACAAGTAGCTG	-	36531
**W39972**	TACTCGAATCAGGATTTTGG	+	39429
**W41797**	TGTGGTGTGTGAACAAGGAAA	-	41254
**42**	CTATCTACCAAAATTTTAACTTCTGGTTCCGGC	-	42381
**W41549**	TATGGCGGTATCCGAAAACA	+	41006
**W54841**	CGGATACGCCTCCATGA	+	54336
**W54225**	TCCTGAAGTTCTTTGTAACGTTGT	+	53720
**69**	CAACCCCTTGGGGTTATCCTGCACTTGGAA	+	135199
		-	81975
**W80924**	TGACGTGGATTGTATCGATTTC	+	80544
**H432**	GGGTTATCCTGCACTTGGAA	+	135209
		-	81975
**W107400**	GCAATAGATGTCTTTCACATACAA	+	105261
**260**	AAGGATTTACCCGGTGTGAG	-	126720
		+	90464
**W94612**	TCCCAAGGGCAGGTTCTTAC	+	124455
		-	92729
**W95255**	CCATCGTTTACGGCTAGGAC	-	123812
		+	93372
**W98266**	CTTATCTCCAGCCCGTGAAC	+	96137
		-	121047
**W100297**	GGTCTCCGCAAAGTCGTAAG	-	98177
		+	119007
**W105011**	CCGTTTCATGAGGATAGACAAA	-	102900
**H401**	GCCAGTCCAATAGCTGCTTC	+	109763
**W18889**	TTCTGCCCTAAGACTATGGATT	+	18208
**W20819**	GGAATGGAAATGAGGGAATG	-	20279
**W19456**	TTCTGACATCTTAGTCTCGTAGTTT	+	18775
**W21210**	CTCGGAATTAGACCATAAGGG	-	20670

**W20900**	GCTTGGCAGAAGAACTTGAGA	+	20360
**W22691**	GTATGCACCAAAAACCTCGG	-	22151
**34**	CTCGGGGATGGAAATGAGGGAATATCTACAAT	+	20275
**156**	AAGATGTGAATTAGGAAGGATTGG	+	21027
**W22358**	TTGCATGGGAACAGATTCAT	+	21818
**W23878**	GAAATACTTAATGCTACGATCTGGG	-	23338
**W58483**	GATTTTAACTTACCCTCTATTTTCG	+	58046
**W60385**	CTACTAATTCGTTGGGTCTACAA	-	59948
**W61165**	CCGACCGTATTTCTCCTGA	+	60708
**W62668**	AGAGATGAACCCAATCCAGAA	-	62212
**W62250**	GCTAGTTGAGTAGTTTTGATTAAGG	+	61777
**W63625**	CCCGAAACAGAATTCCTT	-	63169
**W72808**	TCATCAAGCGGTTCTTATTCG	+	72421
**W74664**	TTCAATGAGTAGTTATAGGCGCA	-	74261
**58**	CTGCCGTATTTATGTTAATGCACTTTCCAATG	+	73468
**W74410**	GCATTCATTTTCTTTGCATCG	+	74007
**313**	CAATTTTTGTTTTTCTTCTAGACGA	-	46416
**W79014**	GGTTTAACAACGGCTACCCA	-	78609
**W5578**	AATGGTATCGAGAATTGATCC	+	5595
**W6851**	CAAGGAGGTATTTTGTGTTTGG	-	7107
**W10898**	CTAATGCGTTCCCCAACG	+	11056
**W12264**	ATAGAAAATGAAACGGTCGACC	-	12426
**W14720**	AATAGTCAAGTACCCCATTTCC	-	14317
**W32396**	GCAAAACTCAGATTGGAGAAGAA	-	31918
**W38034**	ATCTTGTTGCACCCGGTAAC	+	37504
**W39284**	GTCTTACCATGAGCAGATTGTAT	-	38741
**W57280**	TCAGGACTGGGTATCCATGC	+	56831
**W58952**	TGAATTGGCGATCAGAACAC	-	58510
**219**	GGCCTAGTATTTCCGGCAAT	-	58082
**W65036**	CGAACAAATCGAAATGATTGAA	+	64518
**W66044**	CGTTTATTCCCAGATGCTTT	-	65523
**W105880**	ACGTGAAGAGGGAATTGTGC	+	103769
**W11893**	TTTCCAATTAGCTGTTTTTGCAT	+	12056
**W12997**	CATCGTTAGCTTGGAAGGCT	+	13146
**W27717**	CGTCGATTACCAAAAACAAGC	+	27208
**W30641**	TTGGTTGAAAGGCCTGAAAG	+	30159
**W36871**	GACGTTTCCGCCTATATTCC	+	36387
**W38591**	GAAAGAGCCACGGGCTTAT	-	38048
**W51430**	TACCAAAATAGGAATAAGGCTTG	+	50972
**W53119**	CCCTTGTCAGAGATATCTTAACAT	-	52653
**W99809**	GTCCATCGACTACGCCTTTC	-	119495
		+	97689
**W119602**	TGCGGTGACGATACTGTAGG	+	117519
		-	99665
**W9275**	CTGTTCAATTGCGGCCTTAT	-	9433
**W80090**	TCCACATCCCTTTAGTTTTGC	+	79683

**W82197**	TCAAGGCAGTGGATTGTGAA	+	81816
		-	135368
**98**	ATGAAGCTTATCCCCCATCGTCTC	-	119904
		+	97276
**W80234**	GGATCTCGACAATACGAAGCA	-	79823
**W104124**	GCAAGTCTCCGTATCTTATTGG	-	115169
		+	102013
**W115355**	ATCCCGCCATGAGAAG	+	113264
**W16232**	GATTCGACTTCCCCTAGGG	-	15546
**276**	ACCATTAAAGCAGCCCAAGC	-	18086
**W17778**	AGAATGCAAGTCCTCCCCTT	+	17035
**W23499**	TGAGCAAGAGGCTTCAAGAAA	+	22959
**W25382**	ATTGTATGGGAAATACTTCAAGAA	-	24842
**W31373**	CAGCAAGTCAGGGAAAAAGC	-	30900
**W46254**	TAGCAAATTACTGTGGTATGTAAAA	-	45806
**W48442**	GGATAGGTGCAGAGACTCAATG	+	47943
**W50280**	AGATAACCAACCCTGCTGCA	-	49821
**W59953**	AACAAATGTCTGGAACTTTGGA	+	59516
**W61817**	TCCTTATTTCTATCTCATGCAAA	-	61361
**242**	GGAGATGCAGAAATAGTACTTCAAGA	-	61166
**130**	GTATTTCTAATAAGTTGTTTAATAGTTGG	-	68318
**W67189**	GGCTCGTATTTTATCTTTCTTACCA	+	66749
**W73004**	AAATAGGGAAGAGAAAAGTCAAG	+	72603
**W76846**	GGGTGATGCTTTTCTTGAGC	+	76439
**W78260**	GCAATAGTGTCCTTGCCCAT	-	77854
**253**	GCATTGTCATCATATCGTATTATCA	-	78134
**66**	AGGGCATTTCCCATTGATATAGGAACTTTT	-	83018
		+	134156
**W81925**	GGTAGAATGCTAGATGCCCG	+	81544
		-	135640
**W77590**	ATTTTGACCCTATCCCCCAT	-	77187
**W3692**	GGCTCTATCCATTTATTCACTAGAC	-	3695
**W-4**	TTGTTTACGAAGAAATAATAACAA	+	2519
**W-5**	CAGATCGGTTAAATTTCACTG	-	4068
**W34488**	ACCGATATTTTAGCAACAAATCTAA	+	33999
**W36012**	TGCTCGTATTATAGGTCTTGGTGA	-	35528
**W-6**	TGAATACCGATTTACATTTCCG	+	35250
**174**	GAGATGGCCGAGTGGTTGAAGGCG	-	45628
**W44580**	CCTTTGTATGCTCTCCATTGC	+	44024
**W45797**	GCGTAACGTACCTTATGCAA	+	45331
**W47393**	GCATTACAAATGCGATGCTC	-	46918
**W46864**	CAGACCATAATGAAAACGCAAT	+	46398
**W49145**	TGAAAATCCTCGTGTCACCA	-	48779
**W48828**	CCTCTATCCCCAAACCCTCT	+	48392
**W50130**	AGCACATTGGGAACGTAAAT	+	49671
**W51362**	CCCGATTCATAACTAGAAAGC	-	50904

**W-7**	CTATCACATCGAACCCGTTC	-	55988
**W64314**	TTTCTTCTCCTCTTATTCCATATGG	+	63823
**W65536**	AAAACAAACGCGCTACCAAG	-	65007
**128**	CCACGTTCTACCGAACTGAACTAAGAGC	-	64812
**231**	TATAATAGGTAGGGATGACAGGA	-	64959
**W67341**	GAAACTCCAACCAGAATTTAAGA	-	66901
**W-8**	TGATCATATATGTATTCCAATAAGG	+	65394
**W69005**	CCATAAGCTTTGGCTTCGTC	-	68579
**W69829**	AAGCTACCCATTGCATATTG	+	69428
**W71184**	GAATTGTGAGAGCGGACGTT	-	70779
**W70884**	GGGATCAGGGATACTTTCAGC	+	70479
**W71750**	CGAATCTATGGAAGCATTGG	-	71352
**W72170**	GGCTACACAAACCGTTGAAGA	-	71750
**W122891**	TGCTTATTTTCAACTCCCCG	+	120812
		-	96372
**W118236**	TCGTGAAAAGTGATACCTGTGAA	+	116141
		-	101040
**W137891**	TCTCACCATCCCCATAGTGT	-	83106
		+	134078
**W92021**	CGAATTGTTTGTTGACACGG	+	90155
		-	127029
**W93455**	ATGAGAGAAGCACAGAGGTCA	-	91605
		+	125578
**W128213**	ATGATCCGATCGATTGC	+	91322
		-	125865
**W92518**	ATCCTTACTGTCCCTCTACAGAA	-	90652
		+	126529
**W130055**	ATGGCAAGTGCTCTTCCTTG	+	89467
		-	127717
**W125083**	TACCACTGAGCTAATAGCCCG	+	122763
		-	94420
**W126193**	GCCGACACTGACACTGAGAG	+	93312
		-	123872
**W124160**	CCCGAAAAGGAGTCTATTGA	-	95099
		+	122085
**W125409**	GCTGGATCACCTCCTTTTCA	+	94096
		-	123088
**W118951**	CTGGTCGTAGGTTCGAATCC	-	116868
		+	100316
**W109967**	CCGATAAATCCATGGGATA	-	107812
**W111122**	GATAGAGCCATGCTGCG	-	108963
**W-9**	GAACAAAATCTGGATAGATACCGA	+	107390
**W110391**	TCAATCTTTCCAAATCTAATCC	+	108236
**W112816**	CCTAGTAGAAGAACAAATCCCC	+	110659
**W-10**	AGTGGTATCGAGAATTGATCC	+	5595
**W-11**	TAAGGAGGTATTTTGTCTTTGG	-	7107

**W-14**	GGGGTTGTATGATATATCAGGC	+	31706
**W-15**	CAATATATAGAATTCCGGAATTG	-	32778
**W31723**	GCCATTTTGGGGATTCCTAC	+	31250
**W33791**	CTGCTGCAGGACAAGCTGTA	-	33315
**W-16**	GTGGACTAGCACTTGATTTCG	+	55074
**W56293**	CTTGGCAGCATTCCGAGTA	-	55844
**W56344**	AGGGGCTGCAGTAGCTG	-	55895
**W55204**	CAGCTCTAACTCGATTATTTCCTAA	+	54699
**W76331**	TGGATTCTTCAATACCTACTATTGT	-	75926
**W-17**	GCCGTCAATGATTGGTGAGC	+	74640
**W-18**	GCATCTATAGGATAACTTCCATC	+	75655
**W116493**	CGATGCGAAAGTAATTATGC	-	114411
**W-19**	GTAAAGAGTTTACACTGCTTATG	+	112950
**W115989**	TTACATAAAGCTCTTGCTTTGATA	+	113907
**W-12**	TCCTACGAAAGCATAACAGAAAA	-	15341
**W-13**	TTCTGACACCTTAACGCATAGTTT	-	18775
**W16206**	CATCTCTCTTTCAAGGAGGCA	+	15520
**W8579**	TTAAAAGATAGGCTTTGAAATAGG	+	8742
**W10070-1**	GGAGCAATGAACCTATTTGAAG	-	10228
**W10070**	GGAGCAATGAACCTATTTGAAG	+	10228
**W11523**	ACTCAGCCATCTCTCCACAG	-	11686
**W26767**	CGGATCCTCAAGGACAAATG	-	26258
**W25894**	GCTCCGATGGAAACTAGATCA	+	25345
**W84777**	CGTTTTCATTTACCATCTCATG	+	84368
		-	132814
**W88291**	GAAGCCCAAAGAGGTGCATA	-	86413
		+	130771
**W18055**	CCGGTTGGAATCATAAAGTG	-	17314
**W71818**	TCTTCTTCCGAGAACCACCT	+	71420
**W111245**	ATTTGCTTGGCTTTACATCC	+	109086
**W112337**	TTCTAAGTATTTCTTATTGTCGAGC	-	110180
**W111787**	TTTCTATTCATCTTAATGTTCCG	-	109627
**W-20**	TTCAATTGGTTAGAGCACCG	+	16076
**W-21**	TTCTGCCCTAAGACTCTGGATG	-	18208
**W133811**	TGGGATCGTTTGATCGATTT	-	85692
		+	131492
**W135950**	GGGTCGCTTCTTATGGACT	+	85030
		-	132155
**W-23**	TAACGTGAATTGTATCGATTTC	-	80544
**W-22**	AAGATCTTCTTTTCTCCATCTC	+	16693

*Primer sequences mentioned with both a "+" and "-" strand position are located in the Inverted Repeat region.
