# Supplementary material for: The Chloroplast Phylogenomics and Systematics of Zoysia (Poaceae)
Source: Plants (Basel). 2021 Jul 24;10(8):1517. doi: 10.3390/plants10081517 (PMC8400354; doi:10.3390/plants10081517)
Supplement: Supplementary file 1 [file plants-10-01517-s001.zip › plants-1272299-supplementary/Table S3. The Primer list of sequencing of Zoysia macrostachya.pdf]

Table S3. The Primer list of sequencing of *Zoysia macrostachya*

Primer ID	Sequence(5'-3')	Strand*	Position
**256**	CTGGGGAGGTCCGTTTGATA	+	135042
		-	82236
**W81925**	GGTAGAATGCTAGATGCCCG	+	81588
		-	135690
**W137891**	TCTCACCATCCCCATAGTGT	-	83150
		+	134128
**W-3**	AATCCACTGCCTTGATCCAC	-	135501
		+	81855
**H414**	GGGGCGCTTCCGGTTCTGTT	+	82810
		-	134468
**W-2**	TTCTCGCTATTTGGAATGGA	-	84163
		+	133115
**18**	GTCTGTAAATACTGCATATTTGATTCCATC	+	83758
		-	133556
**340**	TTGGAACGAGCATGAAGAAA	-	84968
		+	132310
**W84777**	CGTTTTCATTTACCATCTCATG	+	84412
		-	132864
**W133811**	TGGGATCGTTTGATCGATTT	-	85736
		+	131542
**W88291**	GAAGCCCAAAGAGGTGCATA	-	86458
		+	130820
**W135950**	GGGTCGCTTCTTATGGACT	+	85074
		-	132205
**H422**	CTAAAGAGCGTGGAGGTTCG	-	131106
		+	86172
**H423**	GGTCTTCCTCCACTAGCAGG	+	130181
		-	87097
**99**	GTAGATGCTACATAGTTGGTTCTCAT	+	129316
		-	87956
**106**	TTGGGTTCATTGATATTCCTGGTATAG	+	86834
		-	130437
**H424**	TTTCGACCTTGGTTCCGTAG	+	87809
		-	129469
**W90942**	AATAAGGTTTGATCCTATTCATGG	+	128153
		-	89121
**W92518**	ATCCTTACTGTCCCTCTACAGAA	-	90697
		+	126578
**W130055**	ATGGCAAGTGCTCTTCCTTG	+	89512
		-	127078
**W92021**	CGAATTGTTTGTTGACACGG	+	90200
		-	127029
**W93455**	ATGAGAGAAGCACAGAGGTCA	-	91650
		+	125627

**W94612**	TCCCAAGGGCAGGTTCTTAC	+	124455
		-	92774
**W128213**	ATGATCCGATCGATTGC	+	91367
		-	125914
**H427**	ACCTTGACGTGGTGGAAGTC	+	92392
		-	124886
**H428**	AAGGGGCATGATGACTTGAC	+	123472
		-	93806
**W95255**	CCATCGTTTACGGCTAGGAC	-	123861
		+	93417
**W125083**	TACCACTGAGCTAATAGCCCG	+	122812
		-	94465
**W124160**	CCCGAAAAGGAGTCTATTGA	-	95144
		+	122134
**W125409**	GCTGGATCACCTCCTTTTCA	+	94141
		-	123137
**H429**	CGCATCTTCACAGACCAAGA	+	94864
		-	122414
**W122891**	TGCTTATTTTCAACTCCCCG	+	120861
		-	96417
**10**	CGAACTGCTGCTGAATCCATGGGCAGGCAAG	+	96470
		-	120796
**W100297**	GGTCTCCGCAAAGTCGTAAG	-	98222
		+	119056
**W99809**	GTCCATCGACTACGCCTTTC	-	119544
		+	97734
**W119602**	TGCGGTGACGATACTGTAGG	+	117568
		-	99710
**96**	GTAACTATAACGGTCCTAAGGTAGC	+	98344
		-	118929
**W104124**	GCAAGTCTCCGTATCTTATTGG	-	115218
		+	102115
**W118951**	CTGGTCGTAGGTTCGAATCC	-	116917
		+	100361
**W105880**	ACGTGAAGAGGGAATTGTGC	+	103814
**W107400**	GCAATAGATGTCTTTCACATACAA	+	105306
**W108711**	TCTCAGTGGTTAATGATGCATG	-	106613
**W108608**	AAGCACAATAACTACGCCAAG	+	106510
**W109967**	CCGATAAATCCATGGGATA	-	107857
**W110557**	TAATTCTAACTCCCACATGATAAAA	-	108447
**W-9**	GAACAAAATCTGGATAGATACCGA	+	107435
**W111122**	GATAGAGCCATGCTGCG	-	109008
**W112176**	CGATTCATTTCAATCTGAGGA	+	110127
**W114051**	TTCGGTTTGATAACCTGCTA	-	112002
**W112816**	CCTAGTAGAAGAACAAATCCCC	+	110708
**W116493**	CGATGCGAAAGTAATTATGC	-	114460

**W-19**	GTAAAGAGTTTACACTGCTTATG	+	112999
**W98266**	CTTATCTCCAGCCCGTGAAC	+	96182
		-	121096
**W111245**	ATTTGCTTGGCTTTACATCC	+	109131
**W113787**	TTGATCCATTCTCATCCTGG	+	111738
**W114453**	CGCTTCCGAATTGATCTCAT	+	112400
**103**	GATATCATATTCATGGAATATGATTC	+	131024
		-	86248
**14**	TAGCCCCAGAATGTTTCAGTTTATGCTCCT	-	128711
		+	88557
**339**	AACTGCAAAATCCGATCCAA	+	127510
		-	89768
**W-1**	CGAGATCCACCCTACAATA	+	127379
		-	89900
**97**	ATATAGCTCAGTTGGTAGAGCTCCG	-	95313
		+	121960
**11**	CATTTCGCTCGCCGCTACTACGGGAATCGC	+	120713
		-	96555
**100**	GGGCGAGGTCTCTGGTTCAAGTCCAGGA	+	95209
		-	122061
**W98861**	CTATCGGTCACCCAGGAGTATT	+	120495
		-	96781
**98**	ATGAAGCTTATCCCCCATCGTCTC	-	119953
		+	97321
**95**	TCGTGCGGGTCGGAACTTACCCGAC	+	118894
		-	98379
**9**	GCCGACCGCTCTACCACTGAGCTACTGAGG	+	116877
		-	100391
**W-24**	CTGTAGAGAATTGAGAATTTTCATG	+	101324
		-	115949
**W-25**	CGTGAGACATATAATCGTCGC	-	104205
**H409**	GGTGAAATTGGTAGACACGC	-	106069
**W-26**	GAATTTGACCTTCGCTTTATCTCC	+	105004
**W110391**	TCAATCTTTCCAAATCTAATCC	+	108236
**W-27**	GAATTAGCCAATAAATTGGCTAT	-	109475
**H401**	GCCAGTCCAATAGCTGCTTC	+	109808
**W112922**	CTGACTAAAACTAAAAGATTCAAGG	-	110814
**W115117**	AAATTAATAAGCTGTTTTGTTTCAC	-	113076
**W115989**	TTACATAAAGCTCTTGCTTTGATA	+	113956
**W-4**	TTGTTTACGAAGAAATAATAACAA	+	2520
**W-5**	CAGATCGGTTAAATTTCACTG	-	4067
**24**	CGCCGCTTTAGTCCACTCAGCCATCTCTCC	-	8119
**25**	CTGGGACGGAAGGATTCGAACCTCCGAATAGC	+	6821
**W13690**	AGAACGAATCACACTTTTACCAC	-	13842
**W-12**	TCCTACGAAAGCATAACAGAAAA	-	15346
**W17778**	AGAATGCAAGTCCTCCCCTT	+	17040

**W-13**	TTCTGACACCTTAACGCATAGTTT	-	18779
**309**	TCTTGCATTTATTGCTACTGCAC	+	17151
**W18889**	TTCTGCCCTAAGACTATGGATT	+	18212
**W20819**	GGAATGGAAATGAGGGAATG	-	20282
**W27367**	CCGACCTTGTACGATCCC	+	25861
**W29456**	CAATACCAATTCCTTTTATTCCA	-	28992
**W27717**	CGTCGATTACCAAAAACAAGC	+	27211
**175**	CTTGGGGATTCTTGATTGGTGCTG	-	29810
**W30641**	TTGGTTGAAAGGCCTGAAAG	+	30183
**W31373**	CAGCAAGTCAGGGAAAAAGC	-	30924
**W34488**	ACCGATATTTTAGCAACAAATCTAA	+	34022
**W36012**	TGCTCGTATTATAGGTCTTGGTGA	-	35551
**40**	CCTTGGCTAAACCTTGGAAATCGTAATGC	-	40139
**165**	CATTACCAAGATTAGCACGG	+	165
**43**	GCCCACTATTGGCCAAACCACCTGGGCACTAG	+	42088
**246**	CCTTCCCTTAGAACCGTACTTG	-	43249
**H458**	TGAGCCTTGGTATGGAAACC	-	47831
**W47393**	GCATTACAAATGCGATGCTC	-	46944
**224**	CCATGCATAAACTAAACCAACAA	+	50729
**W53119**	CCCTTGTCAGAGATATCTTAACAT	-	52677
**w55203**	ACAGCTCTAACTCGATTATTTCCT	+	54722
**W-7**	CTATCACATCGAACCCGTTC	-	56007
**W-16**	GTGGACTAGCACTTGATTTCG	+	55099
**w56135**	AGACCCTGTCGTTGTGAGAA	+	55705
**W56293**	CTTGGCAGCATTCCGAGTA	-	55893
**W74410**	GCATTCATTTTCTTTGCATCG	+	74027
**W76331**	TGGATTCTTCAATACCTACTATTGT	-	75946
**W-17**	GCCGTCAATGATTGGTGAGC	+	74660
**W79014**	GGTTTAACAACGGCTACCCA	-	78629
**W-23**	TAACGTGAATTGTATCGATTTC	-	80586
**69**	CAACCCCTTGGGGTTATCCTGCACTTGGAA	+	135249
		-	82019
**W80090**	TCCACATCCCTTTAGTTTTGC	+	79704
**W105376**	GACCAGAAGCAAGCAAGAGG	-	13310
**W117523**	AATAAAAGAATGTACCCTTCTATCC	+	101794
		-	115479
**W-35**	GTGATAAATTCATATGAATTTATGGA	+	102749
**W-36**	GAATAGACAGGTGAACTGAGAATA	-	104376
**W1683**	ATTCACCAGGTCATCAATACG	+	1695
**W-28**	GATTGCAAACCCCTCAGATA	-	2904
**W3004**	TGCTGATACATTAGAAAAATTAAA	+	3016
**395**	CTTCGGGATCGAACATCAAT	-	4644
**36**	CCCATCGTCTAGTGGTTTAGGACATCTCTC	+	15503
**W18757**	ACCATTAAAGCAGCCCAAGC	-	18090
**W16206**	CATCTCTCTTTCAAGGAGGCA	+	15525
**W-20**	TTCAATTGGTTAGAGCACCG	+	16081

**W-29**	GCACCCAGATTTGAACTGGGGAT	+	19048
**W21210**	CTCGGAATTAGACCATAAGGG	-	20673
**W31653**	TATGACGAGGTTGCCTGACA	-	31204
**145**	GATTGGCTGTAGGGCTTGCTTCT	-	33290
**W-14**	GGGGTTGTATGATATATCAGGC	+	31729
**321**	TGCTTAGTCTAGCTTTTATGGAAGC	-	33410
**W39284**	GTCTTACCATGAGCAGATTGTAT	-	38762
**W44580**	CCTTTGTATGCTCTCCATTGC	+	44044
**W-30**	GAGTGGTTTTATTCAAATTCAAAGC	+	43066
**W48442-1**	GGATAGGTGCAGAGACTCAATG	-	47966
**W48442**	GGATAGGTGCAGAGACTCAATG	+	47966
**W49145**	TGAAAATCCTCGTGTCACCA	-	48802
**235**	TAGGGCTATACGGACTCG	-	52009
**W51430**	TACCAAAATAGGAATAAGGCTTG	+	50993
**212**	CAGGAGCAGGGTCGGTCAAAT	-	53934
**W52686**	CCAAAGTGTTTCAAAGGAGGA	+	52244
**H437**	CATGAGTTGTAGGGAGGGAT	-	55734
**W55204**	CAGCTCTAACTCGATTATTTCCTAA	+	54752
**W56293-1**	CTTGGCAGCATTCCGAGTA	-	55863
**W56344**	AGGGGCTGCAGTAGCTG	-	55914
**W80234**	GGATCTCGACAATACGAAGCA	-	79844
**W-33**	GCTCCTATGACTCGAATACACATC	+	78325
**W80924**	TGACGTGGATTGTATCGATTTC	+	80586
**W81925-1**	GGTAGAATGCTAGATGCCCG	-	81588
		+	135690
**W947**	GAAACAGGCTCACGAATACCA	+	947
**72**	CCCGTTGCCTTACCACTTGGCCACGCCCCA	-	6863
**W4463**	TCAAAGTTCTTTCTTTATCTTTAAA	+	4474
**W-21**	TTCTGCCCTAAGACTCTGGATG	-	18212
**276**	ACCATTAAAGCAGCCCAAGC	-	18086
**W23499**	TGAGCAAGAGGCTTCAAGAAA	+	22962
**W25894-1**	GCTCCGATGGAAACTAGATCA	-	25348
**W32396**	GCAAAACTCAGATTGGAGAAGAA	-	31918
**W41129**	CGACTGCATTTTCCAACTGA	-	40607
**W41797**	TGTGGTGTGTGAACAAGGAAA	-	41254
**W45138**	TGTGGTGTGTGAACAAGGAAA	-	41275
**W47393-1**	GCATTACAAATGCGATGCTC	-	46944
**W-31**	TCTAGGCATAATTCCCAATCC	+	45047
**W-32**	GGTATGAGTACTGCGCCGAACATAAACT	-	50076
**W48828**	CCTCTATCCCCAAACCCTCT	+	48415
**320**	TAGCCTTTGTTTGGCAAGCT	-	7390
**W7487**	TGGATCTGTGCGGTAAGGAAAA	+	7764
**W-11**	TAAGGAGGTATTTTGTCTTTGG	-	7108
**W114453-1**	CGCTTCCGAATTGATCTCAT	-	112400
**W48828-1**	CCTCTATCCCCAAACCCTCT	-	48415

**374**	CATCTCGGAAATATTCAGCCA	-	54165
**W54841**	CGGATACGCCTCCATGA	+	54360
**W55204-1**	CAGCTCTAACTCGATTATTTCCTAA	-	54723
**W-34**	CTCAACGGATAAAAGTTACT	-	98858
**W5430**	CTATGTCGAGCCAAGAGCATT	-	5441
**W25382**	ATTGTATGGGAAATACTTCAAGAA	-	24845
**W80234-1**	GGATCTCGACAATACGAAGCA	+	79844
**W33791**	CTGCTGCAGGACAAGCTGTA	-	33338
**W34156**	GCCCTCTTCCTTCCCG	-	33692
**W-10**	AGTGGTATCGAGAATTGATCC	+	5596
**71**	GTTGCTTTCTACCACATCGTTTCAAACGAA	+	5526

*Primer sequences mentioned with both a “+” and “-” strand position are located in the Inverted Repeat region.
