# Supplementary material for: The Chloroplast Phylogenomics and Systematics of Zoysia (Poaceae)
Source: Plants (Basel). 2021 Jul 24;10(8):1517. doi: 10.3390/plants10081517 (PMC8400354; doi:10.3390/plants10081517)
Supplement: Supplementary file 1 [file plants-10-01517-s001.zip › plants-1272299-supplementary/Table S4. The Primer list of sequencing of Zoysia sinica.pdf]

Table S4. The Primer list of sequencing of *Zoysia sinica*

Primer ID	Sequence(5'-3')	Strand*	Position
**256**	CTGGGGAGGTCCGTTTGATA	+	135010
		-	82210
**W81925**	GGTAGAATGCTAGATGCCCG	+	81562
		-	135658
**W137891**	TCTCACCATCCCCATAGTGT	-	83124
		+	134096
**W-3**	AATCCACTGCCTTGATCCAC	-	135391
		+	81829
**H414**	GGGGCGCTTCCGGTTCTGTT	+	82784
		-	134436
**W-2**	TTCTCGCTATTTGGAATGGA	-	84137
		+	133083
**18**	GTCTGTAAATACTGCATATTTGATTCCATC	+	83686
		-	133524
**340**	TTGGAACGAGCATGAAGAAA	-	84942
		+	132278
**W84777**	CGTTTTCATTTACCATCTCATG	+	84386
		-	132832
**W133811**	TGGGATCGTTTGATCGATTT	-	85710
		+	131510
**W88291**	GAAGCCCAAAGAGGTGCATA	-	86431
		+	130771
**W135950**	GGGTCGCTTCTTATGGACT	+	85048
		-	132173
**H422**	CTAAAGAGCGTGGAGGTTCG	-	131075
		+	86145
**H423**	GGTCTTCCTCCACTAGCAGG	+	130150
		-	87070
**99**	GTAGATGCTACATAGTTGGTTCTCAT	+	129285
		-	87929
**106**	TTGGGTTCATTGATATTCCTGGTATAG	+	86807
		-	130406
**H424**	TTTCGACCTTGGTTCCGTAG	+	87782
		-	129438
**W90942**	AATAAGGTTTGATCCTATTCATGG	+	128122
		-	89094
**W92518**	ATCCTTACTGTCCCTCTACAGAA	-	90670
		+	126547
**W130055**	ATGGCAAGTGCTCTTCCTTG	+	89485
		-	127735
**W92021**	CGAATTGTTTGTTGACACGG	+	90173
		-	127047
**W93455**	ATGAGAGAAGCACAGAGGTCA	-	91623
		+	125596

**W94612**	TCCCAAGGGCAGGTTCTTAC	+	124473
		-	92747
**W128213**	ATGATCCGATCGATTGC	+	91340
		-	125883
**H427**	ACCTTGACGTGGTGGAAGTC	+	92365
		-	124855
**H428**	AAGGGGCATGATGACTTGAC	+	123441
		-	93779
**W95255**	CCATCGTTTACGGCTAGGAC	-	123830
		+	93390
**W125083**	TACCACTGAGCTAATAGCCCG	+	122781
		-	94438
**W124160-1**	TCAATAGACTCCTTTTCGGG	+	122103
		-	95117
**W125409**	GCTGGATCACCTCCTTTTCA	+	94114
		-	123106
**H429**	CGCATCTTCACAGACCAAGA	+	94837
		-	122383
**W122891**	TGCTTATTTTCAACTCCCCG	+	120830
		-	96390
**10**	CGAACTGCTGCTGAATCCATGGGCAGGCAAG	+	96443
		-	120765
**W100297**	GGTCTCCGCAAAGTCGTAAG	-	98195
		+	119025
**97**	ATATAGCTCAGTTGGTAGAGCTCCG	-	95286
		+	121929
**98**	ATGAAGCTTATCCCCCATCGTCTC	-	119922
		+	97294
**W99809**	GTCCATCGACTACGCCTTTC	-	119513
		+	97707
**W119602-1**	CCTACAGTATCGTCACCGCA	+	117537
		-	99683
**96**	GTAACTATAACGGTCCTAAGGTAGC	+	98317
		-	118898
**9**	GCCGACCGCTCTACCACTGAGCTACTGAGG	+	116846
		-	100364
**94**	GAAGGTCTCGGCGAGACGAGCCG	+	99296
		-	117921
**W118236**	TCGTGAAAAGTGATACCTGTGAA	+	116159
		-	101058
**W119602**	TGCGGTGACGATACTGTAGG	+	117537
		-	99683
**W104124-1**	GCAAGTCTCCGTATCTTATTGG	+	115187
		-	102031

**W118951**	CTGGTCGTAGGTTCGAATCC	-	116886
		+	100334
**W105880**	ACGTGAAGAGGGAATTGTGC	+	103787
**W107400-1**	GCAATAGATGTCTTTCACATACAA	-	105279
**W107400**	GCAATAGATGTCTTTCACATACAA	+	105279
**W108711**	TCTCAGTGGTTAATGATGCATG	-	106586
**W108608**	AAGCACAATAACTACGCCAAG	+	106483
**W109967**	CCGATAAATCCATGGGATA	-	107830
**W110557**	TAATTCTAACTCCCACATGATAAAA	-	108420
**W-9**	GAACAAAATCTGGATAGATACCGA	+	107408
**W111122**	GATAGAGCCATGCTGCG	-	108981
**W112176**	CGATTCATTTCAATCTGAGGA	+	110038
**W114051**	TTCGGTTTGATAACCTGCTA	-	111971
**W112816**	CCTAGTAGAAGAACAAATCCCC	+	110677
**W116493**	CGATGCGAAAGTAATTATGC	-	114429
**W-19**	GTAAAGAGTTTACACTGCTTATG	+	112968
**W98266**	CTTATCTCCAGCCCGTGAAC	+	96155
		-	121065
**W104124**	GCAAGTCTCCGTATCTTATTGG	-	115187
		+	102031
**82**	CTATGGAGAAATTGGTAGACACGCTGCTCT	-	106038
**W111245**	ATTTGCTTGGCTTTACATCC	+	109104
**W113787**	TTGATCCATTCTCATCCTGG	+	111707
**W114453**	CGCTTCCGAATTGATCTCAT	+	112369
**15**	GCTGTTAATACGAATAGAGAAACTCTGT	-	88613
		+	128598
**H426**	CCCTATTGTTCCGATGGAGA	+	128440
		-	88780
**259**	AAAAACCAAACTCTGCCTTACG	+	126604
		-	90614
**W130741**	GAACAATAGGGCCGTTATGC	+	88789
		-	128431
**11**	CATTTCGCTCGCCGCTACTACGGGAATCGC	+	120682
		-	96528
**100**	GGGCGAGGTCTCTGGTTCAAGTCCAGGA	+	65182
		-	122030
**W98861**	AATACTCCTGGGTGACCGATAG	-	96754
		+	120464
**W-24**	CTGTAGAGAATTGAGAATTTTCATG	+	101297
		-	115918
**W-25**	CGTGAGACATATAATCGTCGC	-	104178
**W110391**	TCAATCTTTCCAAATCTAATCC	+	108254
**W-27**	GAATTAGCCAATAAATTGGCTAT	-	109448
**W115989**	TTACATAAAGCTCTTGCTTTGATA	+	113925
**395**	CTTCGGGATCGAACATCAAT	-	4642

**W4052**	CAAAATAAACAGATCGGT	+	4058
**23**	TTAAAAGCCGAGTACTCTACCGTTGAGTTA	+	3889
**276**	ACCATTAAAGCAGCCCAAGC	-	18088
**W-22**	AAGATCTTCTTTTCTCCATCTC	+	16695
**W31365**	TTTTGATGCAGCAAGTCAGG	+	30894
**W32396**	GCAAAACTCAGATTGGAGAAGAA	-	31920
**W34488**	ACCGATATTTTAGCAACAAATCTAA	+	34001
**W36012**	TGCTCGTATTATAGGTCTTGGTGA	-	35530
**251**	GTAGCCCAAACAAGATGTCCA	-	38175
**W36871**	GACGTTTCCGCCTATATTCC	+	36389
**43**	GCCCACTATTGGCCAAACCACCTGGGCACTAG	+	42068
**246**	CCTTCCCTTAGAACCGTACTTG	-	43230
**H458**	TGAGCCTTGGTATGGAAACC	-	47809
**W47393**	GCATTACAAATGCGATGCTC	-	46919
**W49145**	TGAAAATCCTCGTGTCACCA	-	48780
**224**	CCATGCATAAACTAAACCAACAA	+	50709
**235**	CCATGCATAAACTAAACCAACAA	-	51986
**W56293**	CTTGGCAGCATTCCGAGTA	-	55840
**W56135**	AGACCCTGTCGTTGTGAGAA	+	55682
**244**	AAAACAAACGCGCTACCAAG	+	65003
**W67341**	GAAACTCCAACCAGAATTTAAGA	-	66897
**W76331**	TGGATTCTTCAATACCTACTATTGT	-	75923
**W-17**	GCCGTCAATGATTGGTGAGC	+	74637
**253**	GCATTGTCATCATATCGTATTATCA	-	78131
**W76846**	GGGTGATGCTTTTCTTGAGC	+	76436
**W18889**	TTCTGCCCTAAGACTATGGATT	+	18210
**W-20**	TTCAATTGGTTAGAGCACCG	+	16078
**W20819**	GGAATGGAAATGAGGGAATG	-	20281
**76**	AATCCACTGATTTCTGCCGCTTCCGTTATTGC	+	33232
**40**	CCTTGGCTAAACCTTGGAAATCGTAATGC	-	40119
**165**	CATTACCAAGATTAGCACGG	+	165
**127**	GAATGACCCAGTATCGAATACTGGTAA	+	62906
**231**	TATAATAGGTAGGGATGACAGGA	-	64959
**W65536**	AAAACAAACGCGCTACCAAG	-	65003
**237**	AAGTATTTTACGATTAAGAAG	-	67254
**238**	CGCTCTGTAGGATTTGAACCTAC	+	64766
**129**	GCTCTGTAGGATTTGAACCTACGACATC	+	64767
**58**	CTGCCGTATTTATGTTAATGCACTTTCCAATG	+	103787
**W71818**	TCTTCTTCCGAGAACCACCT	+	71416
**69**	CAACCCCTTGGGGTTATCCTGCACTTGGAA	+	135217
		-	81993
**W80090**	TCCACATCCCTTTAGTTTTGC	+	79680
**W105376**	GACCAGAAGCAAGCAAGAGG	-	103283
**W117523**	GGATAGAAGGGTACATTCTTTTATT	-	115448
		+	101767
**W-35**	GTGATAAATTCATATGAATTTATGGA	+	102722

**W-36**	GAATAGACAGGTGAACTGAGAATA	-	104349
**W1683**	ATTCACCAGGTCATCAATACG	+	1694
**W-28**	GATTGCAAACCCCTCAGATA	-	2903
**24**	CGCCGCTTTAGTCCACTCAGCCATCTCTCC	-	8118
**25**	CTGGGACGGAAGGATTCGAACCTCCGAATAGC	+	6821
**320**	TAGCCTTTGTTTGGCAAGCT	-	7390
**W6509**	TTTTGTGTCGTGCAGAGC	+	6771
**W7136**	GCGTTTGTTTGGCAAGC	+	7391
**W-29**	GCACCCAGATTTGAACTGGGGAT	+	19046
**W21210**	CTCGGAATTAGACCATAAGGG	-	20672
**145**	GATTGGCTGTAGGGCTTGCTTCT	-	33269
**W-14**	GGGGTTGTATGATATATCAGGC	+	31708
**321**	TGCTTAGTCTAGCTTTTATGGAAGC	-	33389
**W48442-1**	GGATAGGTGCAGAGACTCAATG	-	47944
**218**	TTCCTTCCCTTAGAACCGTACTTGAGAG	-	43228
**W46864**	CAGACCATAATGAAAACGCAAT	+	46390
**W73004-1**	AAATAGGGAAGAGAAAAGTCAAG	-	72600
**W77590**	ATTTTGACCCTATCCCCCAT	-	77184
**W80234**	GGATCTCGACAATACGAAGCA	-	79820
**W-33**	GCTCCTATGACTCGAATACACATC	+	78302
**W947**	GAAACAGGCTCACGAATACCA	+	947
**W4463**	TCAAAGTTCTTTCTTTATCTTTAAA	+	4472
**W5430**	CTATGTCGAGCCAAGAGCATT	-	5440
**W-10**	AGTGGTATCGAGAATTGATCC	+	5594
**W-11**	TAAGGAGGTATTTTGTCTTTGG	-	7108
**W33791**	CTGCTGCAGGACAAGCTGTA	-	33317
**W-15**	CAATATATAGAATTCCGGAATTG	-	32780
**H465**	AGTGGTTCAAGGCGTAGCAT	-	45639
**W47393-1**	GCATTACAAATGCGATGCTC	-	46919
**W-31**	TCTAGGCATAATTCCCAATCC	+	45028
**W48828**	CCTCTATCCCCAAACCCTCT	+	48393
**W50130**	AGCACATTGGGAACGTAAAT	+	49672
**W-32**	GGTATGAGTACTGCGCCGAACATAAACT	-	50056
**W51430**	TACCAAAATAGGAATAAGGCTTG	+	50973
**212**	CAGGAGCAGGGTCGGTCAAAT	-	53911
**374**	CATCTCGGAAATATTCAGCCA	-	54142
**W52686**	CCAAAGTGTTTCAAAGGAGGA	+	52221
**W71184-1**	GAATTGTGAGAGCGGACGTT	+	70775
**W72808**	TCATCAAGCGGTTCTTATTCG	+	72418
**W78260**	GCAATAGTGTCCTTGCCCAT	-	77851
**W81925-1**	GGTAGAATGCTAGATGCCCG	-	81562
**W-23**	TAACGTGAATTGTATCGATTTC	-	80562

*Primer sequences mentioned with both a “+” and “-” strand position are located in the Inverted Repeat region.
