# Supplementary figures and images for: The Chloroplast Phylogenomics and Systematics of Zoysia (Poaceae)
Source: Plants (Basel). 2021 Jul 24;10(8):1517. doi: 10.3390/plants10081517 (PMC8400354; doi:10.3390/plants10081517)

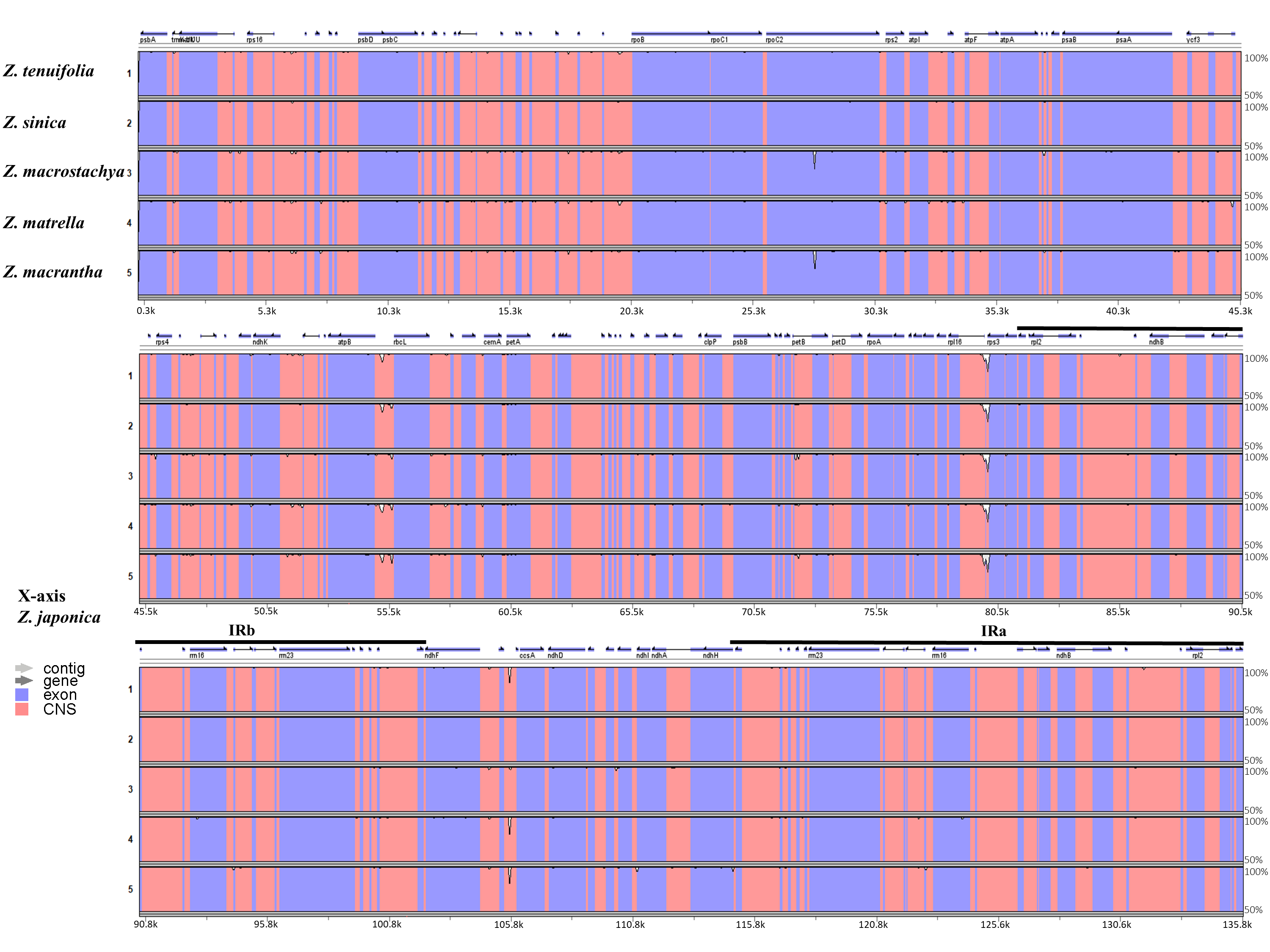

Supplement: Supplementary file 1 [file plants-10-01517-s001.zip › plants-1272299-supplementary/Figure S1. mVISTA identity plot comparing Zoysia plastomes.tif]
